# Human ES-derived MSCs correct TNF-α-mediated alterations in a blood–brain barrier model

**DOI:** 10.1186/s12987-019-0138-5

**Published:** 2019-07-01

**Authors:** Shujun Ge, Xi Jiang, Debayon Paul, Li Song, Xiaofang Wang, Joel S. Pachter

**Affiliations:** 1 Blood-Brain Barrier Laboratory, Dept. of Immunology, UConn Health, 263 Farmington Ave, Farmington, CT 06030 USA; 2 ImStem Biotechnology, Inc., 400 Farmington Ave., Farmington, CT 06030 USA; 3 Present Address: Perelman School of Medicine at University of Pennsylvania, Philadelphia, PA 19104 USA

**Keywords:** Blood brain barrier, Brain endothelial cells, Mesenchymal stem/stromal cells, Tight junction, MS and EAE

## Abstract

**Background:**

Immune cell trafficking into the CNS is considered to contribute to pathogenesis in MS and its animal model, EAE. Disruption of the blood–brain barrier (BBB) is a hallmark of these pathologies and a potential target of therapeutics. Human embryonic stem cell-derived mesenchymal stem/stromal cells (hES-MSCs) have shown superior therapeutic efficacy, compared to bone marrow-derived MSCs, in reducing clinical symptoms and neuropathology of EAE. However, it has not yet been reported whether hES-MSCs inhibit and/or repair the BBB damage associated with neuroinflammation that accompanies EAE.

**Methods:**

BMECs were cultured on Transwell inserts as a BBB model for all the experiments. Disruption of BBB models was induced by TNF-α, a pro-inflammatory cytokine that is a hallmark of acute and chronic neuroinflammation.

**Results:**

Results indicated that hES-MSCs reversed the TNF-α-induced changes in tight junction proteins, permeability, transendothelial electrical resistance, and expression of adhesion molecules, especially when these cells were placed in direct contact with BMEC.

**Conclusions:**

hES-MSCs and/or products derived from them could potentially serve as novel therapeutics to repair BBB disturbances in MS.

**Electronic supplementary material:**

The online version of this article (10.1186/s12987-019-0138-5) contains supplementary material, which is available to authorized users.

## Background

Multiple sclerosis (MS) and its animal model, experimental autoimmune encephalomyelitis (EAE), are inflammatory, demyelinating disorders of the central nervous system (CNS) that ultimately culminate in axonal loss and permanent neurological disability [1–3]. In both conditions, immune cell trafficking into the CNS is widely considered to contribute to pathogenesis, yielding characteristic multifocal perivascular infiltrates predominantly comprised of lymphocytes and monocytes/macrophages [4–8]. Disruption of the blood–brain barrier (BBB)—a possible cause and/or consequence of neuroinflammation—is also a hallmark of these pathologies [9, 10] and a potential target of therapeutics [11–14]. A compromised BBB could additionally thwart rehabilitative efforts in MS by dysregulating the homeostatic milieu necessary for endogenous neural repair [15].

Despite the wide spectrum of disease modifying therapies (DMTs) to treat MS, the mechanisms of action of most are immunomodulatory and immunosuppressive in nature, and current DMTs are mainly effective on the inflammatory facets of the disease [16–18]. Few MS therapeutics are directed toward neuroprotection and/or repair of CNS tissue, or recovery of BBB integrity and function [19–22]. However, significant promise on this latter front comes from vast reports on the use of mesenchymal stem/stromal cells (MSCs) to modify the course of EAE [23–38]. MSCs are but one type of many unspecialized stem cells that can either replicate as undifferentiated cells or differentiate into other cell types in the body [39, 40]. Among the diverse cell lineages MSCs can assume are bone, cartilage and fat [41], and there are a variety of sources from which MSCs are routinely isolated [42, 43], including bone marrow (BM), adipose tissue, amniotic fluid, dental pulp, umbilical cord, menstrual fluid, peripheral blood and synovial membranes. MSCs may also be derived from human embryonic stem cells (hESCs) [44, 45]—with hES-MSCs showing superior therapeutic efficacy compared to BM-MSCs in an EAE setting [37]. Because MSCs exhibit both immunomodulatory and reparative effects [46–48], and support neuroregeneration [49–55], it is conceivable MSCs can remediate disruption of the BBB in MS.

Supporting this possibility, several reports have described the ability of MSCs to inhibit and/or repair damage to the BBB or related blood-spinal cord barrier (BSCB) in other animal models of neurological disease, including stroke [56, 57], intracerebral hemorrhage [58], intracerebral LPS injection [59], MPTP toxicity [60] and chronic spinal cord injury [61]. And virally transduced, interferon β-secreting MSCs, when co-administered intravenously with the anti-inflammatory drug minocycline, attenuated the clinical severity of EAE while suppressing BSCB disruption [36]. This combinatorial therapy further resulted in an increase, within spinal cord tissue, of occludin—a major transmembrane protein component of the specialized tight junction (TJ) complexes that contribute to the BBB [62–65]. However, it has not yet been reported whether, in an inflammatory milieu, MSCs alone directly influence brain microvascular endothelial cells (BMECs) that comprise the BBB.

Experiments were therefore conducted to determine if hES-MSCs—which were previously shown to exert prophylactic as well as therapeutic effects in EAE [37]—could reverse alterations in a murine BMEC, BBB model [66–68] that were induced by TNF-α, a pro-inflammatory cytokine expressed in the perivascular inflammatory milieu during EAE [69, 70]. Additionally, comparison was made between the effects of direct contact of hES-MSCs with BMEC versus those achieved when both cell types were separated by a filter. hES-MSCs were observed to correct TNF-α-induced changes in TJ proteins, permeability, transendothelial electrical resistance, and expression of adhesion molecules, with performance being superior when these cells were placed in direct contact with BMEC. Results indicate hES-MSCs and/or products derived from them could potentially serve as novel therapeutics to repair BBB disturbances in MS.

## Methods

### Animals

C57BL/6 mice were obtained from the Charles River Laboratories, Inc. (Wilmington, MA) and used as the source for cultured BMEC. Mice were sacrificed by CO_2_ inhalation following the Animal Care and Use Guidelines of the University of Connecticut Health Center (Animal Welfare Assurance A3471-01) and approved protocol 101618-0620.

### Cell culture Mouse brain microvascular endothelial cells (BMECs)

Bulk microvessels [71] were first prepared from brains of C57BL/6 mice, age approximately 4–6 weeks, and BMEC derived from these vessels using immuno-bead selection as previously elaborated by this laboratory [66, 68]. Freshly isolated cells were grown in DMEM/F12 containing 10% plasma-derived horse serum, 10% fetal bovine serum (FBS), 1% antibiotic–antimycotic (all from GIBCO BRL, Rockville, MD), 100 μg/ml heparin, and 100 μg/ml endothelial cell growth supplement (BD Biosciences, Bedford, MA) to confluence in 35-mm plates coated with collagen IV (BD Biosciences) and passaged only one time for experimentation. For all experiments, BMECs were plated onto Transwell filter inserts (Costar, Cambridge, MA). In the Transwell format, the top chamber (T) reflects the luminal side, and the bottom chamber (B) the abluminal side of the endothelium in vivo. TNF-α was applied to both the top and bottom Transwell chambers for 24 h to provoke changes in BMEC before addition of hES-MSCs, and remained in the cultures for an additional 24 h in absence or presence of hES-MSCs. Any return to normal BMEC parameters (i.e., without TNF-α) following hES-MSC addition was considered a reversal.

### Human embryonic stem cell-derived mesenchymal stem/stromal cells (hES-MSCs)

Mesenchymal stem cells were derived by ImStem Biotechnology Inc. from human embryonic stem cells (hESCs), line ESI-053, via a trophoblast-like intermediate stage, as previously detailed [72]. hES-MSCs were grown in 6-well plates (Costar) coated with 0.1% gelatin (Sigma-Aldrich, St. Louis, MO) and in MSC medium: Minimum Essential Medium Eagle Alpha Modification supplemented with 20% FBS, 1X nonessential amino acids, 2 mM glutamine, and 50 U/ml penicillin/streptomycin (all from GIBCO). hES-MSCs were maintained at 37 °C in a 5% CO_2_ humidified atmosphere. Only hES-MSCs at < 5 passages were used throughout the study. The use of hES-MSCs in this study was approved by the Stem Cell Research Oversight Committee of the University of Connecticut (#2012-005).

### Bone marrow-derived mesenchymal stem/stromal cells (BM-MSCs)

BM‐SC lines #4461 and #4462 were used and derive from fresh BM, as described [37, 72]. As for the hES-MSCs, BM‐MSCs were grown in 6-well plates coated with 0.1% gelatin and in MSC medium. BM‐MSCs were maintained at 37 °C in a 5% CO_2_ humidified atmosphere. Only BM‐MSCs at < 5 passages were used throughout the study. The use of BM‐MSCs in this study was approved by the Stem Cell Research Oversight Committee of the University of Connecticut (#2012-005).

### bEND.3 cells

Immortalized cell line bEND.3, derived from a mouse brain capillary hemangioma [73], was obtained from the American Type Culture Collection (ATCC, Manassas VA) and maintained at 37 °C in a 5% CO_2_ humidified atmosphere. bEND.3 cells were grown in DMEM containing 10% FBS, 2 mM l-glutamine, and 50 U/ml penicillin/streptomycin (all from GIBCO). These cells were used exclusively as a source of protein standard and RNA for CLN-5 determination in Western blotting and qRT-PCR analysis, respectively.

### Permeability assay

BMECs were grown to confluence on Transwell inserts (24-well format, 1.0-μm pore, Costar) coated with collagen IV, and monolayer paracellular permeability was determined as reported by Mark and Davis [74]. After TNF-α treatment, hES-MSCs were added to the top chamber for 24 h. Permeability was measured at 2 h after adding 100 μg/ml fluorescein dextran, Mw_r_ 40,000 (FDX-40000; Molecular Probes, Eugene, OR), in assay buffer (0.1% BSA in DMEM) to the top chamber. Samples (50 μl) were removed from the bottom chamber, and analyzed using a Perkin Elmer 1420 Wallac Victor2 multi-label plate reader with fluorescence-detecting capabilities (excitation λ 488 nm; emission λ 510 nm). A permeability coefficient (PC) for FITC-dextran was determined by the following equation: PC (cm/min) = V/(SA × Cd) × (Cr/T), where V is the volume in the receiver (bottom) chamber (1.5 cm^3^), SA is surface area of the cell monolayer (0.33 cm^2^), Cd is the concentration of marker in the donor chamber at time 0, and Cr is the concentration of marker in the receiver at sampling time T [74]. PC was determined for triplicate samples and a mean value established. Data are reported as x-fold change of mean control PC value ± S.E.

### Trans-endothelial electrical resistance (TEER)

BMECs were grown to confluence on Transwell inserts (12-well format, 1.0-μm pore, Costar) coated with collagen IV (BD Biosciences). Cells were ± treated with TNF-α (Invitrogen), and transendothelial electrical resistance (TEER) measured using a STX2 chopstick electrodes connected to an EVOM2 voltohmmeter (World Precision Instruments, Berlin, Germany). The TEER was measured at 24 h after adding the hES-MSCs. The TEER (Ω × cm^2^) was calculated by subtracting the resistance of a blank membrane from the measured resistance and then multiplying this by the membrane surface area. Data are reported as x-fold change of mean control value (no TNF-α or hES-MSCs) ± S.E.

### Immunostaining

BMECs on Transwell inserts (24-well format, 1.0 µm pore, Costar) coated with collagen IV, and hES-MSCs on 8-well chamber slides coated with 0.1% gelatin, were washed with phosphate-buffered saline, pH 7.4 (PBS; GIBCO) and fixed in 3.7% formaldehyde (Sigma)/PBS for 10 min at room temperature. Fixed cells were then permeabilized by incubation with 0.1% Triton X-100 (Sigma) in PBS for 10 min at room temperature, and blocked with 5% normal goat serum/PBS at 4 °C overnight. Cells were then incubated with a 1:50 dilution of rabbit polyclonal anti-ZO-1 (Cat# 61-7300, Zymed, San Francisco, CA) and occludin (Cat# 71-1500, Zymed) antibody or Alexa Fluor 488-conjugated anti-mouse monoclonal (4C3C2) anti-claudin-5 (CLN-5) (Cat# 352588, Invitrogen, Carlsbad, CA) for 2 h at room temperature, followed by 3 × 10 min washes with PBS. BMECs and hES-MSCs that were exposed to anti-ZO-1 and occludin antibody were next incubated in the dark with a 1:200 dilution of FITC-conjugated goat anti-rabbit IgG (Vector Labs, Burlingame, CA) for 1 h at room temperature, and then washed 3 × 10 min washes with PBS. After completing staining, all inserts were cut out from the Transwells, mounted on glass slides, viewed and photographed under a Zeiss LSM 610 confocal microscope (20× or 40×, 0.5 NA objective). To quantify relative tight junction protein expression, immunofluorescent images were imported into Imaris^®^ suite version 7.1 × 64 software (Bitplane Inc., South Windsor, CT). Relative intensity values corresponding to the level of tight junction immunostaining were measured from 25 randomly chosen areas, each defining 10 × 10 pixels, traced in a non-overlapping manner along junctional regions at sites of intercellular contact, as previously described [67] Mean pixel intensity values were obtained by averaging the values of all pixel intensities in the defined areas. Data are reported as x-fold change of mean control value ± S.E.

### Isolation of extracellular vesicles

hES-MSCs were cultured to confluence. Prior to experimentation, cells were switched to media supplemented with exosome-depleted fetal bovine serum (Exo-FBS™; Systems Biosciences, Mountain View, CA) and grown for an additional 12 h with 10 ng/ml TNF-α to stimulate EV release. Extracellular vesicles (EVs) were then isolated from the hES-MSCs supernatant as recently described [75]. Briefly, the hES-MSCs supernatant was sequentially spun at 300×*g* for 10 min at 4 °C, 2000×*g* for 10 min at 4 °C, 8000×*g* for 30 min at 4 °C to remove whole cells, large cell fragments, and apoptotic bodies, respectively. The clarified supernatant was then spun at 100,000×*g* for 60 min at 4 °C to pellet both exosome and microvesicle EV subtypes. EVs were then extracted in cell lysis buffer (Signosis, Santa Clara, CA) and an aliquot directly subject to qRT-PCR as detailed [76].

### qRT-PCR

Total RNA was extracted from cell cultures using the RNeasy kit (QIAGEN, Mansfield, MA) according to the manufacturer’s instructions. RNA was treated with Turbo DNase (Ambion, Austin, TX) according to the protocol provided by the manufacturer, and cDNA synthesized from total RNA using the SuperScript III first-Strand synthesis system (Invitrogen) standard protocol with random hexamers (Roche, Indianapolis, IN), extension temperature at 42 °C for 60 min. Resulting cDNA was stored at − 80 °C until used for further analysis. Measurements of cDNA levels were performed by qRT-PCR using an ABI PRISM 7500 Sequence Detection System Version 1.3, and SYBR green (ABI) fluorescence was used to quantify relative amplicon amount. RPL-19 was used as reference for relative gene expression. Relative quantification was performed using the 2^−ΔΔ^Ct method of Fleige et al. [77]. RT negative controls and no-template controls showed negligible signals (Ct value > 40). Melting curve analysis was used to ensure reaction specificity. RNA expression is reported as x-fold of control ± S.E. The RNA level from EV is reported as Ct value. Sequences of primers used are indicated in Table 1 and Additional file 1: Table S1.

**Table 1:** List qRT–PCR mouse primer sequences

Gene	Forward (5′–3′)	Reverse (5′–3′)
RPL-19	CGCTGCGGGAAAAAGAAG	CTGATCTGCTGACG GAGTTG
CLN-5	TGCCGCGAACAGTTCCTAC	CCAGCTGCCCTTTCAGGTTA
ZO-1	CTCGGAAAAATGAAGAATATGGTC	CACCATCTCTTGCTGCCAAA
Occludin	GGACTGGGTCAGGGAATATCC	GCAGACCTGCATCAAAATTTCTC
VE-cadherin	CACTGCTTTGGGAGCCTTC	GGGGCAGCGATTCATTTTTCT
ICAM-1	GGTGACTGAGGAGTTCGACAGAA	ACCGGAGCTGAAAAGTTGTAGACT
VCAM-1	GTGACTCCATGGCCCTCACT	CGTCCTCACCTTCGCGTTTA
CCL2	GGCTCAGCCAGATGCAGTTAA	CC GCCTACTCATTGGG TCA
CXCL12	GCTCCTCGACAGATGCCTTG	GACCCTGGCACTGAACTGGA

### Western blotting

bEND.3, hES-MSCs and hES-MSC–derived EVs were solubilized in 8 M urea containing protease inhibitor cocktail (Sigma). Protein concentration was assayed by the Micro BCA protein assay kit (ThermoFisher Scientific, Grand Island, NY). Lysates containing 15 μg of bEND.3, hES-MSC or hES-MSC–derived EV protein were separated by electrophoresis on 4–20% Mini-PROTEAN^®^ TGX™ Precast SDS-PAGE gels and transferred onto PVDF membranes (Bio-Rad Laboratories, Hercules, CA). Membranes were then blocked with 5% bovine serum albumin (BSA) in Tris-buffered saline with Tween-20 (TBST) (ThermoFisher Scientific, Grand Island, NY) for 1 h at room temperature, followed by incubation overnight at 4 °C with the CLN-5 antibody (1:200; Life Technologies, Carlsbad, CA) diluted in 5% BSA in TBST. Following incubation with anti-mouse HRP-conjugated secondary antibody (1:400; Cell Signaling), blots were developed using the chemiluminescent HRP substrate kit (SuperSignal West Pico Chemiluminescent Substrate, ThermoFisher Scientific, Grand Island, NY) and signal detected using a G:Box XX6 digital gel imager (Syngene, Frederick, MD). Images were acquired by GeneSys software (Syngene). Since there is not yet consensus in the literature on an internal loading protein control for extracellular vesicles (EVs), nor a protein generally recognized that is equally present in bEND.3 cells, hES-MSCs, and hES-MSC-derived EVs, a loading “control” was not included. Instead, equal amounts of total protein were loaded.

### Transendothelial migration assay

Both hES-MSCs and BM-MSCs were labeled with red fluorescent membrane dye PKH-26 (Life Technologies, Carlsbad, CA). Briefly, all MSCs were plated on gelatin coated 6-well plates and, after reaching confluence, were trypsinized, spun down, and resuspended in 500 µl DMEM. Cells were then incubated with PKH26 dye (2 × 10^−6^ M) at room temperature for 5 min according to manufacturer’s instructions, followed by washing with PBS.

BMECs were sub-cultured at 2.5 × 10^5^ cells/cm^2^ onto Transwell filter inserts (24-well format, 8.0-μm pore, Costar) that had been previously coated with a hydrated layer of collagen I (BD Bioscience) and IV, as described [78]. PKH26-labeled hES-MSCs and BM-MSCs were added to the top chamber at a density of 1.0 × 10^4^ cells/cm^2^, and chemokine CCL2 (10 nM, Peprotech, Rocky Hill, NJ) added to the bottom chamber for 24 h at 37 °C. After this time, images were first gathered, using an epifluorescence fluorescence microscope (IX-70; Olympus, Tokyo, Japan images), of the hES-MSCs and BM-MSCs that had transmigrated into the bottom chamber. MSCs were then collected from the bottom chamber, stained with 0.4% Trypan blue (Sigma), and counted with the Countess^®^ Automated Cell Counter (Invitrogen).

In separate samples, unlabeled hES-MSCs were introduced to the top chamber of a Transwell containing BMECs and, after 24 h, the filters containing BMECs and attached hES-MSCs were washed with PBS and fixed with 4% paraformaldehyde. Filters were immunostained for CLN-5 and ZO-1 (as described above, “Immunostaining” section), and processed for 3D reconstruction.

### Statistics

Each experiment consisted of 3 replicates (derived from a single preparation of BMECs [or MSCs]) repeated 3 times (each time from a different BMEC preparation), for a total N = 9 samples per group. All statistical analyses were performed employing GraphPad Prism 5 software (La Jolla, CA) and the values were expressed as mean ± standard error (S.E.). Statistical comparisons were performed using a one-way analysis of variance (ANOVA). Results were considered significant at a p ≤ 0.05.

## Results

### TNF-α-induced changes in barrier properties in BMEC

**Fig. 1:**
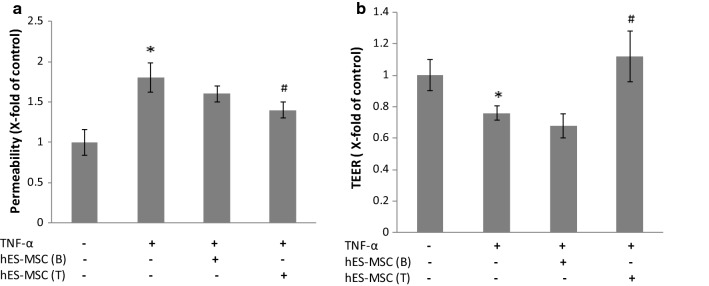
TNF-α-induced changes in barrier properties in BMEC. **a** BMEC monolayer permeability. BMECs were plated on 24-well Transwell inserts, allowed to achieve confluence, and then (±) exposed to 10 ng/mL TNF-α added to both the bottom and top chamber for 24 h at 37 °C. After this time, hES-MSCs were added to either the bottom (B) or top (T) chamber for an additional 24 h. Subsequently, 100 μg/ml fluorescein dextran was added to the top chamber. Samples (50 μl) were removed from the bottom chamber at 2 h and analyzed by plate reader. **b** BMEC monolayer TEER. BMECs were grown to confluence on Transwell filter inserts. Following TNF-α and hES-MSC treatments, TEER measurements were performed. Changes in permeability and TEER following different treatments were reported as x-fold change of control value. Data are presented as mean ± SE. Each experiment consisted of 3 replicates (derived from a single preparation of BMECs) repeated 3 times (each time from a different BMEC preparation), for a total N = 9 samples per group. *p < 0.05 compared with control group, ^#^*p* < 0.05 compared with TNF-α treated group. For purposes of comparison to the literature, absolute mean values of controls for permeability and TEER were 1.2 × 10^−5^ cm/min and 256 Ω cm^2^, respectively

Employing the dual-chamber, Transwell® paradigm, initial experiments sought to gauge whether hES-MSCs could potentially modify changes in BBB integrity that result from a cytokine thought to significantly contribute to the inflammatory milieu in MS/EAE. Specifically, corrective effects of hES-MSCs on BMEC monolayer permeability and transendothelial electrical resistance were measured, and shown in Fig. 1. The hES-MSCs were either applied to the top chamber; i.e., in direct contact with BMEC, or plated in the lower chamber, from which only conditioned media could access the overlying BMEC. TNF-α treatment increased the relative flux of fluorescein dextran (Mw_r_, 40,000), in agreement with prior studies with other BBB models [78–81]. Introduction of hES-MSCs to the top chamber resulted in a statistically significant reversal of TNF-α elevated permeability, while application of hES-MSCs to the bottom chamber did not elicit a similarly significant effect.

TNF-α treatment also caused a reduction in TEER, as previously described with other BBB models [80, 82]. However, direct contact of hES-MSCs with BMEC in the top chamber returned TEER to its normal value, while addition of hES-MSCs to the bottom chamber was again without effect. To discount the possibility that the effect of hES-MSCs might stem from their passively “coating” or “plugging” the BMEC monolayer, hES-MSCs were also added to non-TNFα-stimulated BMECs to see if this resulted in altered TEER. Additional file 2: Fig. S1 shows that 24 h after application of hES-MSCs to resting BMECs, passive attachment of the former to the latter resulted in no significant change in TEER.

### TNF-α-induced changes in junctional protein/gene expression

**Fig. 2:**
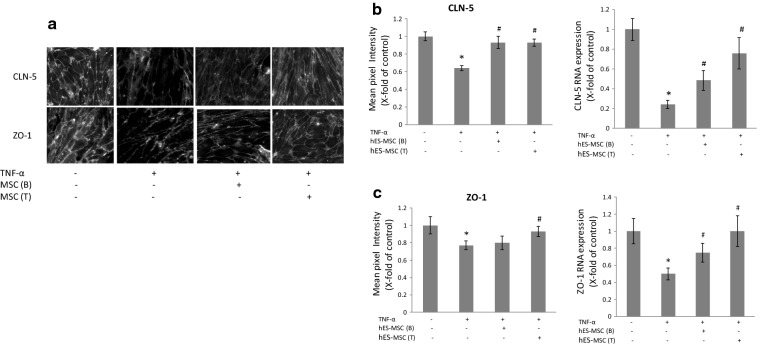
Effects of hES-MSCs on TNF-α-induced changes in TJ protein/gene expression. BMECs were plated on 24-well Transwell inserts, allowed to achieve confluence, and then (±) exposed to 10 ng/ml TNF-α added to both the bottom and top chamber for 24 h at 37 °C. After this time, hES-MSCs were added to either the bottom (B) or top (T) chamber for an additional 24 h. **a** BMECs were fixed with 4% paraformaldehyde, and then immunostained for TJ protein CLN-5 and ZO-1. **b** Relative intensity of CLN-5 and ZO-1 immunostaining. **c** Relative quantification of CLN-5 and ZO-1 mRNA by qRT-PCR. Changes in CLN-5 and ZO-1 following different treatments were reported as x-fold change of control value. Data are presented as mean ± SE. Each experiment consisted of 3 replicates (derived from a single preparation of BMECs) repeated 3 times (each time from a different BMEC preparation), for a total N = 9 samples per group. *p < 0.05 compared with control group, ^#^*p* < 0.05 compared with TNF-α treated group

To determine a possible basis for the TNF-α-induced loss in barrier properties, and their correction by hES-MSCs, quantitative immunofluorescence was performed for several TJ proteins. Figure 2 shows that both claudin-5 (CLN-5), an integral membrane protein that is a critical determinant of the BBB [83] and indispensable for BMEC integrity [84], and ZO-1, a peripheral membrane protein through which CLN-5 links to the actin cytoskeleton [85, 86], decreased in response to TNF-α exposure. These responses occurred by 24 h of cytokine treatment and align with other reports of TNF-α effects on these proteins [87–89]. By contrast, staining intensity of occludin, another integral TJ protein, was not decreased by the same TNF-α treatment (Additional file 2: Fig. S2), and its regulation in BMECs was not analyzed further. Application of hES-MSCs to either the top or bottom chamber reversed the TNF-α effect on CLN-5 staining, both qualitatively and quantitatively (Fig. 2). However, only placement of these cells in the top chamber corrected quantitative effects on ZO-1 staining, noticeable gaps in ZO-1 membrane localization being observed following hES-MSC application to the bottom chamber. Since TJ protein changes had already occurred prior to introduction of hES-MSCs, i.e., by 24 h of TNF-α exposure, these cells appeared to correct the damage to CLN-5 and ZO-1 rather than just prevent it.

Consistent with the reduced staining of CLN-5 and ZO-1 in response to TNF-α, genes encoding both proteins likewise demonstrated reduced expression following cytokine exposure (Fig. 2). Introduction of hES-MSCs to either the top or bottom chamber also reversed the deficit in expression of both genes. Importantly, the probes chosen to detect CLN-5 and ZO-1 in the co-cultures recognized mouse but not human transcripts (Additional file 1: Table S1), and thus reflected de novo gene expression by mouse BMEC and not human hES-MSCs.

Aside from altering expression of CLN-5 and ZO-1 in BMECs, hES-MSCs also demonstrated immunostaining for these same proteins, as well as occludin (Additional file 2: Fig. S3A). The immunostaining patterns of all three TJ proteins were diffuse in the cytoplasm, with a tendency for CLN-5 and occludin to be more concentrated toward the nucleus. Gene expression of CLN-5, ZO-1 and occludin in hES-MSCs was confirmed by qRT-PCR (Additional file 2: Fig. S3B and Additional file 1: Table S1). This was not a general property of MSCs, as BM-MSCs did not show staining or detectable gene expression for CLN-5 or occludin.

**Fig. 3:**
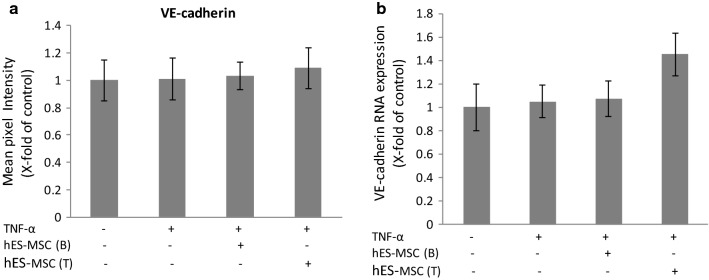
Effects of hES-MSCs on TNF-α-induced changes in protein/gene expression of VE-cadherin. BMECs were plated on 24-well Transwell inserts, allowed to achieve confluence, and then (±) exposed to 10 ng/ml TNF-α added to both the bottom and top chamber for 24 h at 37 °C. After this time, hES-MSCs were added to either the bottom (B) or top (T) chamber for an additional 24 h. **a** Relative intensity of VE-cadherin immunostaining. **b** Relative quantification of VE-cadherin mRNA by qRT-PCR. Changes in VE-cadherin following different treatments were reported as x-fold change of control value. Data are presented as mean ± SE. Each experiment consisted of 3 replicates (derived from a single preparation of BMECs) repeated 3 times (each time from a different BMEC preparation), for a total N = 9 samples per group

In contrast to their effects on TJ components CLN-5 and ZO-1, neither TNF-α (at 10 ng/ml) nor hES-MSCs impacted protein or gene expression of VE-cadherin (Fig. 3), an integral membrane protein of adherens junctions, membrane specializations interspersed with TJs at the BBB [90, 91], and previously shown to be co-dysregulated along with CLN-5 and barrier properties following BMEC exposure to pro-inflammatory chemokine CCL2 [78]. A prior report showing TNF-α-induced downregulation of VE-cadherin in cultured human BMEC [87], might reflect species and/or cell age-dependent differences in cytokine responsiveness, the human cells being of fetal origin.

### hES-MSC-derived extracellular vesicles do not carry CLN-5 protein/mRNA

**Fig. 4:**
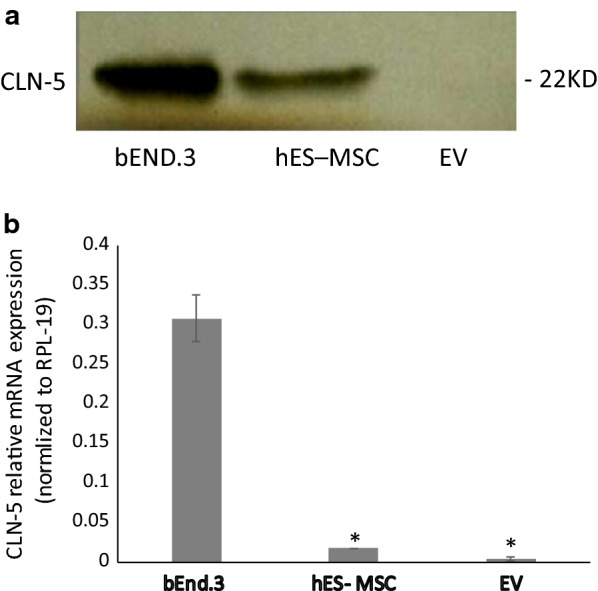
CLN-5 protein/gene expression by hES-MSCs and hES-MSC-derived EVs. hES-MSCs were grown in 6-well plates coated with 0.1% gelatin. Cultures of hES-MSCs or bEnd.3 (as a CLN-5 control) were treated with TNF-α (10 ng/ml) for 24 h and extracted for protein or subject to RNA isolation. EVs were prepared from culture supernatants of hES-MCS. (Top) Western blot for CLN-5 protein. (Bottom) qRT-PCR for CLN-5 mRNA. Because total RNA in EVs was too low to accurately detect, qRT-PCR was performed directly in lysed EV extracts following reverse transcription, as described (76). Data are presented as mean ± SE. Each experiment was repeated 3 times. *p < 0.001 compared with bEnd.3

Because hES-MSCs were observed to express CLN-5, additional studies sought to determine if these cells could potentially employ extracellular vesicles (EVs) to transfer CLN-5 protein or mRNA to BMEC. EVs are nano-size, membrane bound structures shed from numerous cell types—which mediate intercellular communication and can convey a broad spectrum of bioactive molecules (including protein, mRNA, miRNA, and DNA) over long and short distances [92–94]. These cell membrane derivatives are heterogeneous in size and route of derivation. Exosomes are the smallest type EV, generally ranging from 40 to 100 nm in diameter, and derive from multivesicular endosome fusion with the plasma membrane, while microvesicles are typically in the 100–1000 nm range, and arise from exocytotic budding of the plasma membrane [95, 96]. Insofar as MSC-derived EVs have been shown to mediate a variety of therapeutic effects [97–99]—in particular, remediation of vascular-associated brain injury [100–103]—and EVs from several sources carry junctional proteins including CLN-5 [75, 104–107], hES-MSC-derived EVs were analyzed for CLN-5 expression. Western blot analysis in Fig. 4a shows that while hES-MSCs express CLN-5 (though low in comparison to that found in bEND.3s), a preparation of total EVs (containing both exosomes and microvesicles) from these cells did not contain a detectable amount of this protein. This is in contrast to BMEC-derived EVs, which express this protein in both EV subtypes [75]. Also, expression of CLN-5 mRNA was barely detectable in hES-MSC-derived EVs (Fig. 4b).

### TNF-α-induced changes in gene expression of adhesion molecules

**Fig. 5:**
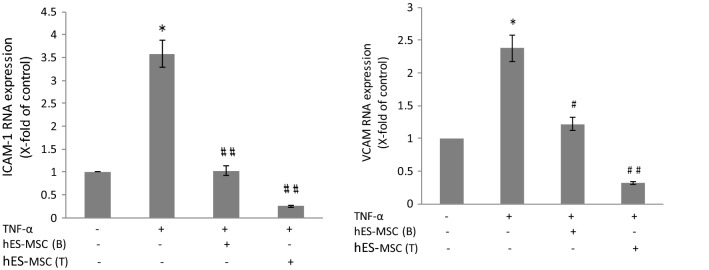
Effects of hES-MSCs on TNF-α-induced changes in gene expression of adhesion molecules. BMECs were plated on 24-well Transwell inserts, allowed to achieve confluence, and then (±) exposed to 10 ng/ml TNF-α added to both the bottom and top chamber for 24 h at 37 °C. After this time, hES-MSCs were added to either the bottom (B) or top (T) chamber for an additional 24 h. Relative quantification of ICAM-1 and VCAM-1 mRNA was determined by qRT-RCR. Changes in adhesion molecule expression following different treatments were reported as x-fold change of control value. Data are presented as mean ± SE. Each experiment consisted of 3 replicates (derived from a single preparation of BMECs) repeated 3 times (each time from a different BMEC preparation), for a total N = 9 samples per group. *p < 0.05 compared with control group; ^#^*p* < 0.01 and ^# #^*p* < 0.001 compared with TNF-α treated group

Expression of adhesion molecules ICAM-1 and VCAM-1 by BMEC is critical for leukocyte attachment and transendothelial migration in vitro and in vivo [108–111] and reflects changes in status of the BBB [112]. Thus, further experiments evaluated whether hES-MSCs could correct any alterations in gene expression of both these adhesions molecules. Figure 5 shows TNF-α treatment caused significant up-regulation of ICAM-1 and VCAM expression by BMEC, reaffirming the inflammatory phenotype induced in these cells by pro-inflammatory cytokine exposure [113–117]. Application of hES-MSCs reversed the up-regulation of both adhesion molecules, and was effective irrespective of being placed in the top or bottom chamber. In fact, placement of hES-MSCs in the top chamber apparently lowered expression of both adhesion molecules to less than their control values.

### TNF-α-induced changes in gene expression of pro-inflammatory chemokines

**Fig. 6:**
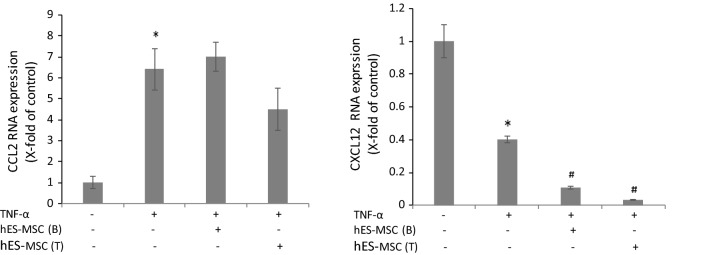
Effects of hES-MSCs on TNF-α-induced changes in gene expression of pro-inflammatory chemokines. BMECs were plated on 24-well Transwell inserts, allowed to achieve confluence, and then (±) exposed to 10 ng/ml TNF-α added to both the bottom and top chamber for 24 h at 37 °C. After this time, hES-MSCs were added to either the bottom (B) or top (T) chamber and incubated for and additional 24 h. Relative quantification of CCL2 and CXCL12 mRNA was determined by qRT-RCR. Changes in chemokine expression following different treatments were reported as x-fold change of control value. Data are presented as mean ± SE. Each experiment consisted of 3 replicates (derived from a single preparation of BMECs) repeated 3 times (each time from a different BMEC preparation), for a total N = 9 samples per group. *p < 0.01 compared with the control group; ^#^*p* < 0.01 compared with TNF-α treated group

Next, hES-MSC effects on TNF-α-induced changes in gene expression of two chemokines associated with neuroinflammation was investigated. Chemokines CCL2/MCP-1 and CXCL12/SDF-1 are both produced by BMEC and their expression and/or localization is altered during MS/EAE in ways to facilitate leukocyte infiltration of the CNS and further BBB compromise [71, 118–121]. Specifically, CCL2 causes disruption of the BBB and reduced inter-endothelial localization of several TJ proteins, including CLN-5 and ZO-1 [67, 68, 78, 119, 122, 123]. And CXCL12 purportedly acts in a negative capacity to retain infiltrating leukocytes in the perivascular space during MS [120, 121]. Figure 6 reveals TNF-α stimulation of BMECs caused up-regulation of CCL2 gene expression, in accord with prior reports of elevated CCL2 release [124] and mRNA level [113, 125] following TNF-α treatment. In opposite fashion, TNF-α stimulation decreased CXCL12 gene expression by BMECs, paralleling the response seen after stimulation with another pro-inflammatory substance: LPS [126]. But, hES-MSCs were not able to correct the altered expression of either chemokine, no matter whether introduced into the top or bottom of the Transwell. Certain TNF-α effects on BMEC are thus refractory to hES-MSC action. This underscores hES-MSCs actually can reverse some effects of TNF-α, and not merely inhibit this cytokine’s interactions with BMEC.

### Transendothelial migration of hES-MSCs

**Fig. 7:**
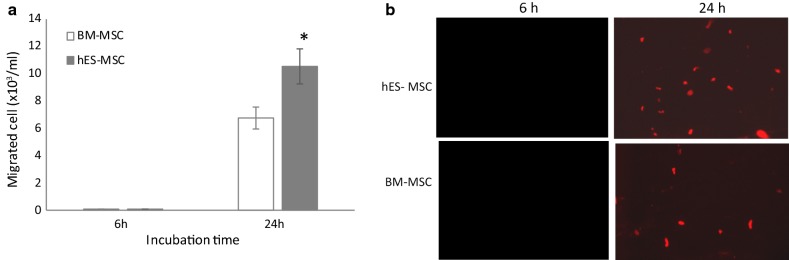
Transendothelial migration of hES-MSCs. BMECs were plated on 24-well Transwell inserts, allowed to achieve confluence, and then PKH-26 labeled hES-MSCs or BM-MSCs were added to top of chamber. **a** After 6 h and 24 h at 37 °C, migrated hES-MSCs and BM-MSCs were collected from the from bottom chamber and counted. **b** Epifluorescence images of hES-MSCs and BM-MSCs that migrated into the bottom chamber at 6 h and 24 h. Data are presented as mean ± SE. Each experiment consisted of 3 replicates (derived from a single preparation of BMECs) repeated 3 times (each time from a different BMEC preparation), for a total N = 9 samples per group. *p < 0.05 compared with BM-MSC group

Lastly, the ability of PKH-26-labeled hES-MSCs to undergo transendothelial migration across BMEC in the presence of exogenous chemokine is shown in Fig. 7. CCL2 was selected as the chemokine, in light of its prominent role(s) in inflammation at the BBB in MS/EAE [119]. Consistent with their superior ability to invade the CNS parenchyma [37], hES-MSCs migrated more efficiently than did BM-MSCs. Thus, while hES-MSCs introduced into the top chamber initially encountered the apical surface of the BMEC monolayer (luminal side in vivo) in the above experiments, it is possible hES-MSCs exerted their effects through interactions on the basolateral surface (abluminal side in vivo). To better appreciate the initial sites of interaction of hES-MSCs with BMECs, co-cultures of these cells were immunostained for TJ proteins CLN-5 and ZO-1, and analyzed by high-resolution confocal microscopy and 3D image reconstruction (Additional file 2: Fig. S4). CLN-5 was chosen to specifically highlight endothelial TJs, and ZO-1 for its ability to more intensely and diffusely label the hES-MSC cytoplasm (see Fig. 2), thus providing resolution of the two cell types. Additional file 2: Fig. S4a shows the BMEC monolayer with its CLN-5-rich intercellular boundaries, and an aggregate of cells attached to the apical surface of the BMECs at what appear to be sites of CLN-5 concentration, perhaps revealing incipient hES-MSC-BMEC interactions prior to paracellular transmigration of hES-MSCs. The concentration of CLN-5 at the site of hES-MSC attachment is spotlighted in a series of individual z-slices that reveal the most intense CLN-5 staining lies in a plane with the cellular aggregate and above that of the rest of the BMEC monolayer (Additional file 2: Fig. S4b). This positioning might reflect redistribution of BMEC membrane to the apical surface to facilitate hES-MSC attachment to, and/or migration through, junctional regions. Though not identified by antibody specific to hES-MSCs, these aggregates were not observed in BMEC cultures alone and, thus, are most likely hES-MSC in origin and not any contaminating pericytes or glial cells.

## Discussion

While MSCs have been heralded as a potential novel therapy for neurodegenerative diseases in general [51, 127–129], and MS in particular [42, 55, 130–133], the repertoire of actions by these cells at the BBB in vivo—which is compromised in these conditions—is unclear. The current experiments highlight several possible routes through which hES-MSCs might act to promote BBB repair. These cells reversed a number of effects induced in BMEC by the prototypical, pro-inflammatory cytokine TNF-α. Specifically, they corrected changes in permeability, protein and gene expression of TJ proteins CLN-5 and ZO-1, and gene expression of adhesion molecules ICAM-1 and VCAM-1. Because the co-cultures evaluated included only BMEC and hES-MSCs, the effects on endothelial tissue were isolated, and removed from potential actions on other components of the neurovascular unit, e.g. astrocytes and pericytes. Moreover, as TNF-α-induced changes occurred by 24 h—prior to introduction of hES-MSCs—the subsequent action(s) by these cells may be interpreted as being corrective or therapeutic, rather than preventative or prophylactic. These findings are in accord with previous reports that MSCs both attenuated BBB/BSCB damage in a variety of animal models of neurologic disease other than MS/EA [56–61], and down-regulated ICAM-1 expression in oxygen-glucose deprived (OGD)/re-oxygenated bEND.3 cells [57], a virally-transformed brain capillary hemangioma cell line [73]. Collectively, our results underscore the potential use of hES-MSCs in a clinical setting to lessen neurovascular damage and restore CNS barrier integrity.

In many cases, the corrective effect of hES-MSCs was dependent on these cells being introduced to the top chamber and, thus, able to make direct contact with BMEC. Other cases showed application of hES-MSCs to either top or bottom chamber was equally effective. In no instance, however, was application of hES-MSCs only to the bottom chamber productive. The need to position hES-MSCs in direct contact with BMEC in order to achieve some effects, likely rules out paracrine signaling, e.g., through the secretion of proteins/peptides, as the exclusive mode of BBB repair. However, as some effects were observed following introduction of hES-MSCs to the bottom chamber, at least partial reparative action would appear to result from release of diffusible factors by these cells. That this is the case with hES-MSC effects on ICAM-1 and VCAM-1 expression is supported by observation by Cheng et al. [57], who reported conditioned media from cultured bone marrow-derived MSCs significantly mitigated ICAM-1 expression induced by OGD/re-oxygenation of bEND.3 cells.

Since hES-MSCs were observed to bind to and migrate across BMEC, this suggests opportunity for these two cell types to engage in physical and/or juxtacrine interactions at the luminal/apical as well as the abluminal/basolateral BMEC surface in vivo. This is in agreement with a previous finding that a large proportion of transmigrated MSCs were retained in the subendothelial space of a similar, rat BMEC-based, BBB model system [134]—perhaps further implying an affinity of MSCs for the BMEC abluminal/basolateral membrane. Migration of hES-MSCs across the BMEC-derived BBB model is also consistent with the former having been shown to penetrate the spinal cord parenchyma following intraperitoneal administration to mice induced to develop EAE [37]. The mechanism by which hES-MSCs cross BMEC is currently unclear, and critical receptor/ligand players in the process have not been established. As BMEC and hES-MSCs express CLN-5, ZO-1, and occludin, one possibility might be transendothelial migration of hES-MSCs exploits the “zipper mechanism,” wherein endothelial junctional contacts are temporarily replaced with homophilic and/or heterophilic interactions between corresponding hES-MSC and endothelial junctional/adhesion proteins [135–137]. Such reasoning would hold that low concentration of TJ proteins at the plasma membrane of hES-MSCs—as reflected in the prominence of cytoplasmic staining (Additional file 2: Fig. S3A—would be necessary for preventing these cells from self-aggregating [138] and, instead, favor forming brief associations with endothelial cells. The observation MSCs can pass non-destructively through transiently-formed, inter-endothelial gaps in an analogous BBB model [134], lends support to this possibility. Extending this argument, the superior transendothelial migration observed for hES-MSCs compared to BM-MSCs might be related to the detectable expression of CLN-5 and/or occludin in the former but not the latter cells.

Migration of hES-MSCs into the CNS could potentially directly impact the parenchymal neural cell population in a variety of regenerative ways [139–141]. But, as demonstrated here, another therapeutic action of hES-MSCs could be to reconstitute physical barrier properties of the endothelium of the BBB. This might occur passively, by whole hES-MSCs plugging “gaps” [142] and/or “integrating” [143] within endothelium, or actively, by inducing structural repair mechanisms—such as amending TJ protein expression—in endothelial cells.

In addition to the action of hES-MSCs, themselves, diffusible derivatives of these cells, e.g., EVs, could also contribute to restoration of the BBB phenotype. Since hES-MSC-derived EVs failed to express CLN-5 protein and displayed only barely detectable amounts of CLN-5/ZO-1 mRNA, it is doubtful such EVs directly convey structural elements or their genetic blueprints and, thus, are unlikely to be the immediate source of heightened CLN-5 protein in TNF-α-stimulated BMEC treated with hES-MSCs. This doesn’t discount the possibility, however, hES-MSC-derived EVs might carry information that regulates restoration of TJ proteins in BMEC, rather than transport these actual proteins or their encoding mRNAs. In fact, MSC-derived EVs have been shown to protect TJ structure in tubular epithelial cells [144] and provide a wide array of neuroprotective and neuroreparative effects [145, 146]. An attractive candidate for mediating this MSC action is miRNA, a prominent component of EVs [147, 148] that serves as a critical regulator of several endothelial junctional proteins [149]. Also, MSC-derived exosomal miRNAs have been reported to resolve wound inflammation [150] and promote functional recovery and neurovascular plasticity after traumatic brain injury [100]. It may further be that EVs responsible for modifying certain aspects of BBB repair are released from hES-MSCs and act in a juxtacrine manner only after these cells make appropriate contact with BMEC, as has been described following leukocyte:endothelial interaction [151]. Soluble factors other than EVs might additionally contribute to TJ repair. The ability of hES-MSCs to correct TNF-α-induced changes in ICAM-1 and VCAM-1 gene expression in BMEC may further help to amend BBB disturbances in vivo, by limiting disruptive leukocyte extravasation through TJs. Future studies are directed at resolving the mechanism(s) by which hES-MSCs restore integrity to the BBB.

## Additional files

**Additional file 1: Table S1.** List qRT-PCR human primer sequences.

**Additional file 2: Fig. S1.** TEER value of BMECs is not passively altered by hES-MSCs. BMECs were cultured on Transwell filters and, following their achieving confluence, hES-MSCs applied, as in Fig. 1 (except no TNF-α was added). After 24 h, TEER was measured. Change in TEER following addition of hES-MSCs is reported as x-fold change of control value. Data are presented as mean ± SE. Each experiment consisted of 3 replicates (derived from a single preparation of BMECs) repeated 3 times (each time from a different BMEC preparation), for a total N = 9 samples per group. No significant difference was detected. **Fig. S2.** Immunostaining of occludin. BMECs were plated on 24-well Transwell inserts, allowed to achieve confluence, and then (±) exposed to 10 ng/ml TNF-α added to both the bottom and top chamber for 24 h at 37 °C. BMECs were fixed with 4% paraformaldehyde, and then immunostained for the TJ protein occludin. **Fig. S3.** TJ protein/gene expression in hES-MSCs and BM-MSCs. **A.** hES-MSCs and BM-MSCs were grown in the 8-well chamber slides coated with 0.1% gelatin. At confluence, hES-MSCs and BM-MSCs were fixed with 4% paraformaldehyde, and then immunostained for TJ proteins CLN-5, ZO-1 and occludin**. B.** hES-MSCs and BM-MSCs, grown as described in **A**, were subect to total RNA extraction for relative measurement of CLN-5, ZO-1 and occludin mRNA by qRT-PCR **(B)**. Data are presented as mean ± SE.. Each experiment consisted of 3 replicates (derived from a single preparation of MSCs) repeated 3 times (each time from a different MSC preparation), for a total N = 9 samples per group. Ct values, and not relative expression values are reported, as Ct values for BM-MSC CLN-5 and occludin mRNA were > 35 and, thus, not considered detectable. *p < 0 0.01 compared with hES-MSC group. **Fig. S4.** Aggregates of hES-MSCs interact with BMEC monolayer. BMECs were plated on 24-well Transwell inserts and allowed to achieve confluence. Thereafter, hES-MSCs were added to top chamber for 24 h, and chemokine CCL2 placed in the bottom chamber. Co-cultures were then fixed with 4% paraformaldehyde, and immunostained for CLN-5 (green) and ZO-1 (red), while nuclei were stained with DRAQ5 (blue). (A, left) Projection image of all three stains reconstructed from a confocal z-series. CLN-5 staining is concentrated at the intercellular junctions of the BMEC monolayer, while ZO-1 is more diffuse and most evident associated with an aggregate of hES-MSCs (designated by the dotted line). (A, right) Projection image highlighting ZO-1 more clearly shows the aggregate of hES-MSCs, along with lesser intense staining of BMEC junctions. (B) Individual z-slices showing the aggregate of ZO-1-stained, hES-MSCs cells at the apical BMEC surface. At this level, a dense area of CLN-5 staining (arrows), projecting upward from the apical surface, is seen surrounding one side of the aggregate. Images are representative of three experiments.

## Data Availability

If needed, more information on the results presented can be obtained via the corresponding authors upon reasonable request.

## Abbreviations

BBB: blood–brain barrier
BSCB: blood-spinal cord barrier
BM: bone marrow
BMEC: brain microvascular endothelial cell
BM-MSC: Bone marrow-derived mesenchymal stem/stromal cell
CNS: central nervous system
CLN-5: claudin-5
DMT: disease modifying therapy
EAE: experimental autoimmune encephalomyelitis
EV: extracellular vesicles
FBS: fetal bovine serum
hESC: human embryonic stem cell
hES-MSC: Human embryonic stem cell-derived mesenchymal stem/stromal cell
MS: multiple sclerosis
MSC: mesenchymal stem/stromal cell
OGD: oxygen-glucose deprived
TJ: tight junction
TEER: transendothelial electrical resistance
