# Supplementary material for: Human ES-derived MSCs correct TNF-α-mediated alterations in a blood–brain barrier model
Source: Fluids Barriers CNS. 2019 Jul 1;16:18. doi: 10.1186/s12987-019-0138-5 (PMC6600885; doi:10.1186/s12987-019-0138-5)
Supplement: Supplementary file 1 — Additional file 1: Table S1. List qRT-PCR human primer sequences. [file 12987_2019_138_MOESM1_ESM.docx]

**Table S1: List qRT–PCR human primer sequences.**

| Gene Forward (5′-3′) Reverse (5′-3′) |
| --- |
| RPL-19 GGG CAT AGG TAA GCG GAA GG TCA GGT ACA GGC TGT GAT ACA  CLN-5 GACTCGGTGCTGGCTCTGAG CGTAGTTCTTCTTGTCGTAG  ZO-1 CGGTCCTCTGAGCCTGTAAG GGATCTACATGCGACGACAA  Occludin TCAGGGAATATCCACCTATCACTTCAG CATCAGCAGCAGCCATGTACTCTTCAC |
