# Supplementary figures and images for: Human ES-derived MSCs correct TNF-α-mediated alterations in a blood–brain barrier model
Source: Fluids Barriers CNS. 2019 Jul 1;16:18. doi: 10.1186/s12987-019-0138-5 (PMC6600885; doi:10.1186/s12987-019-0138-5)

## Slide 1
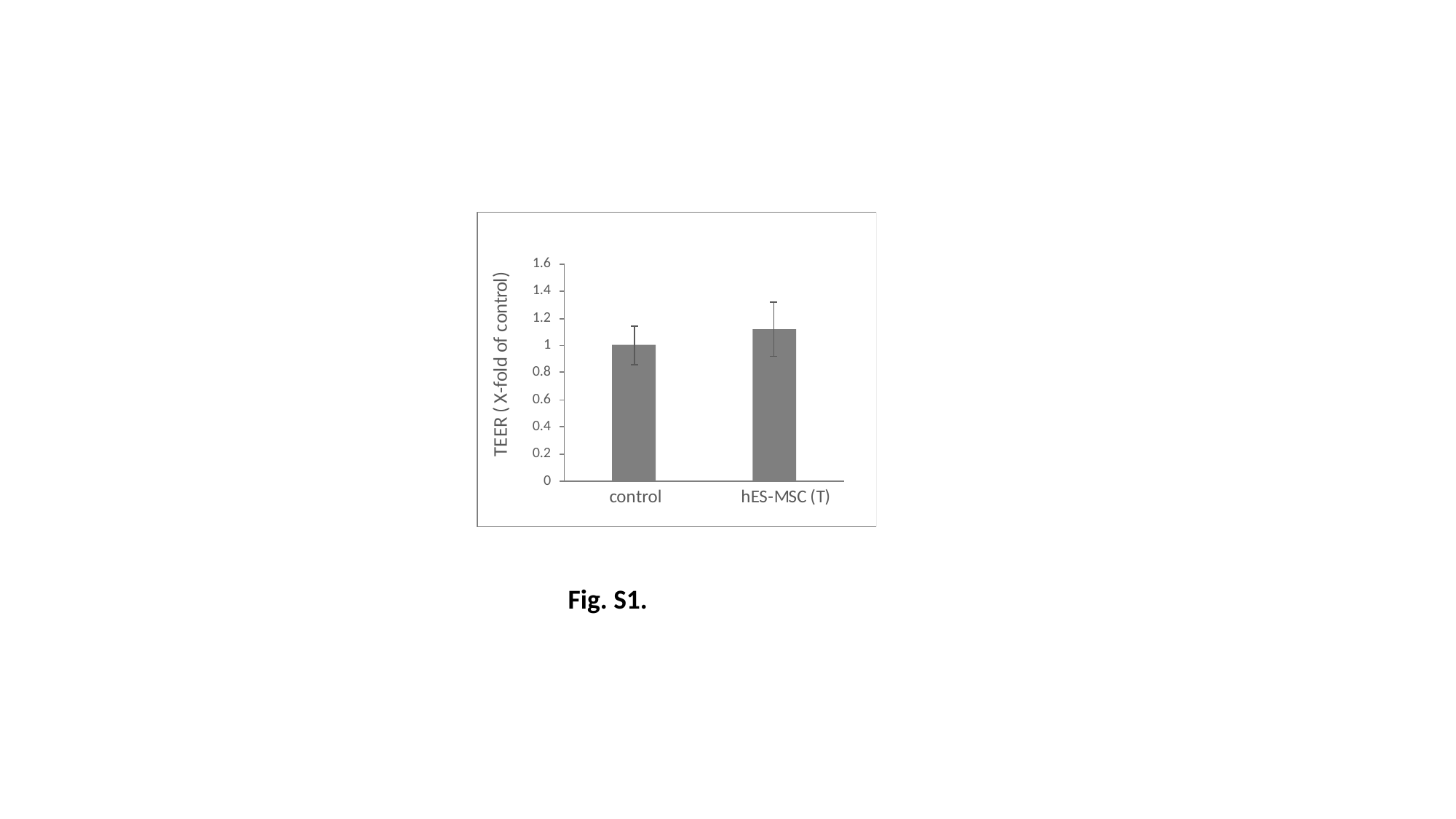

Fig. S1.

## Slide 2
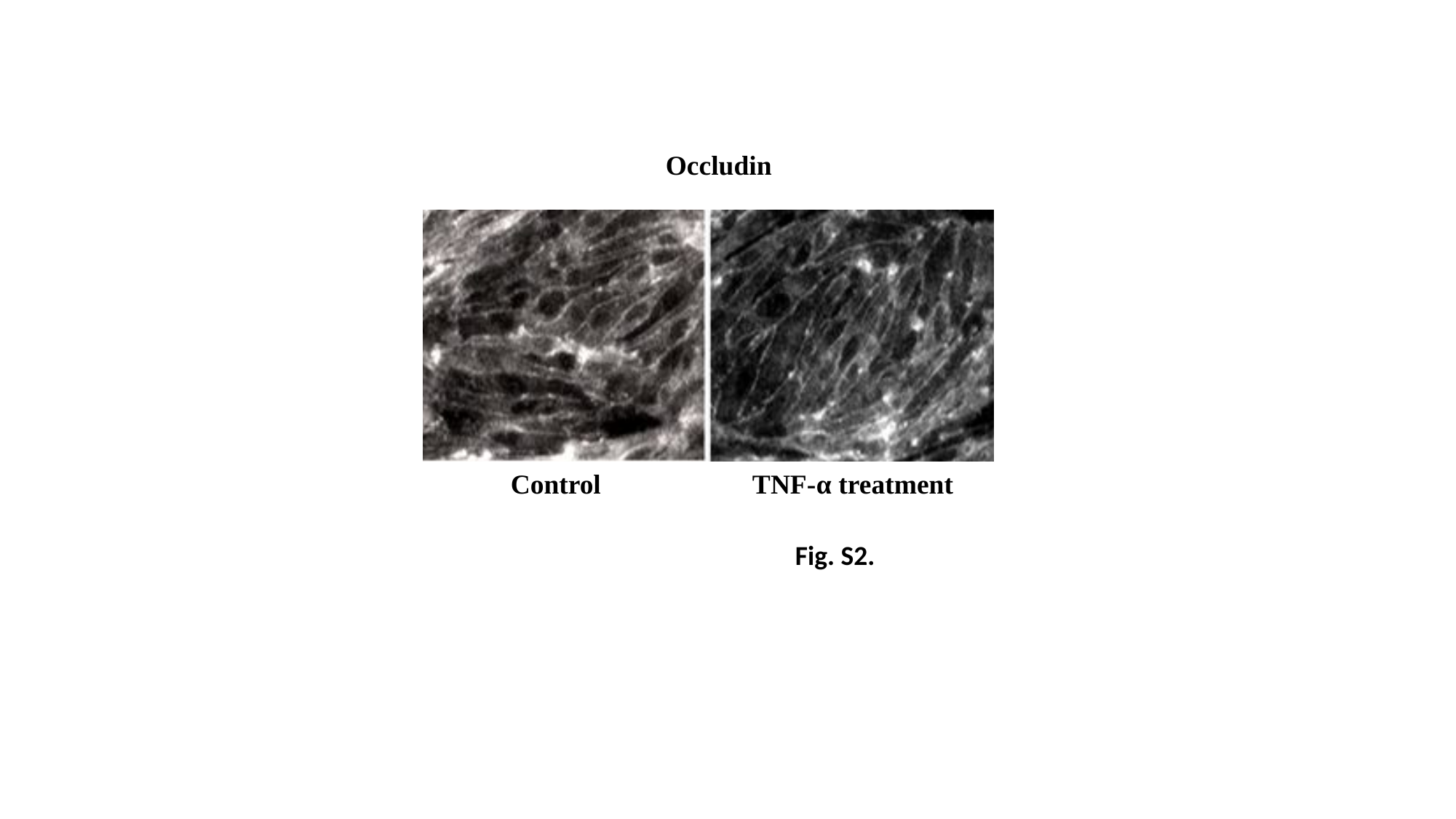

Occludin
 Control TNF-α treatment
Fig. S2.

## Slide 3
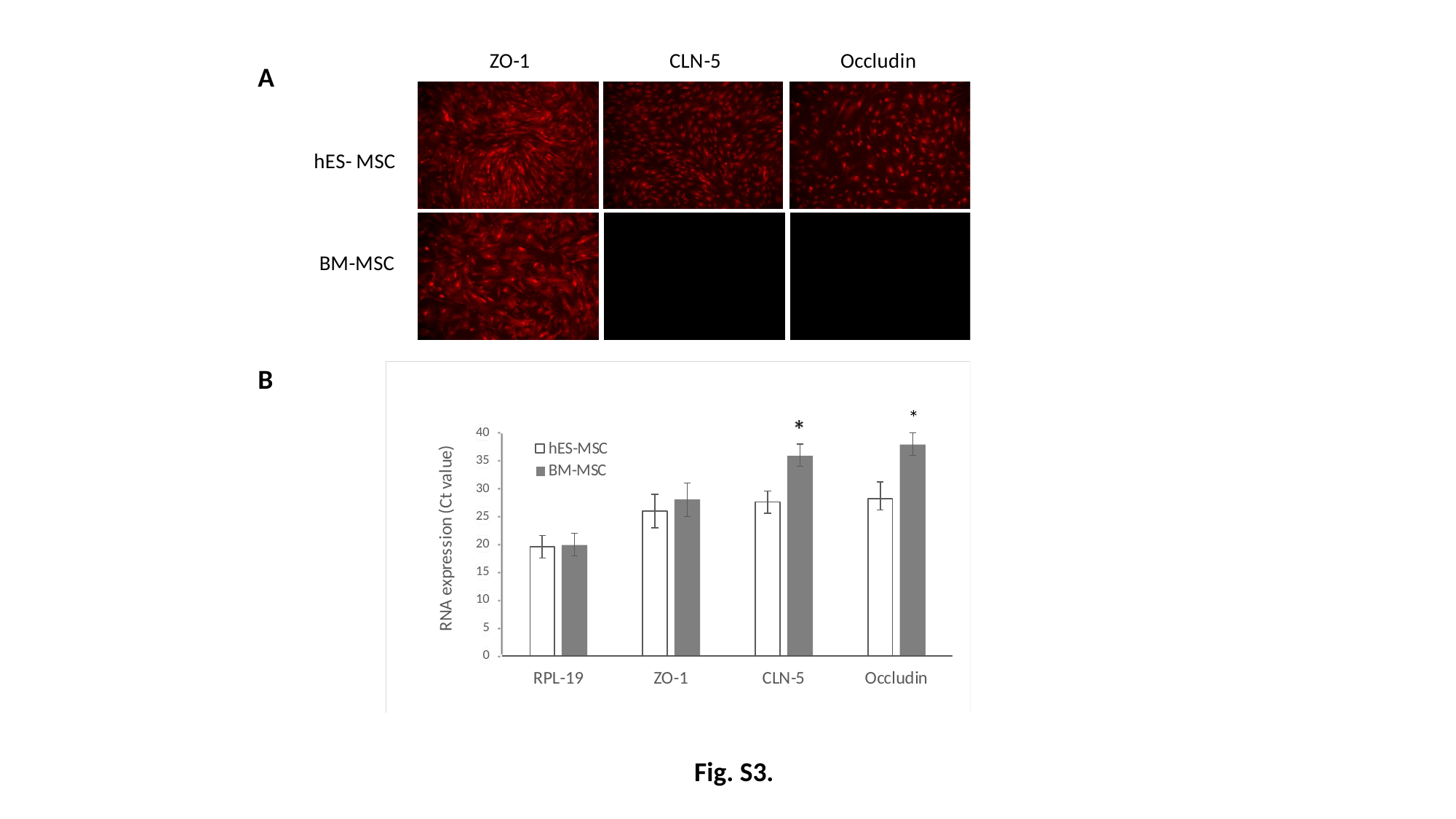

A
B
Fig. S3.

## Slide 4
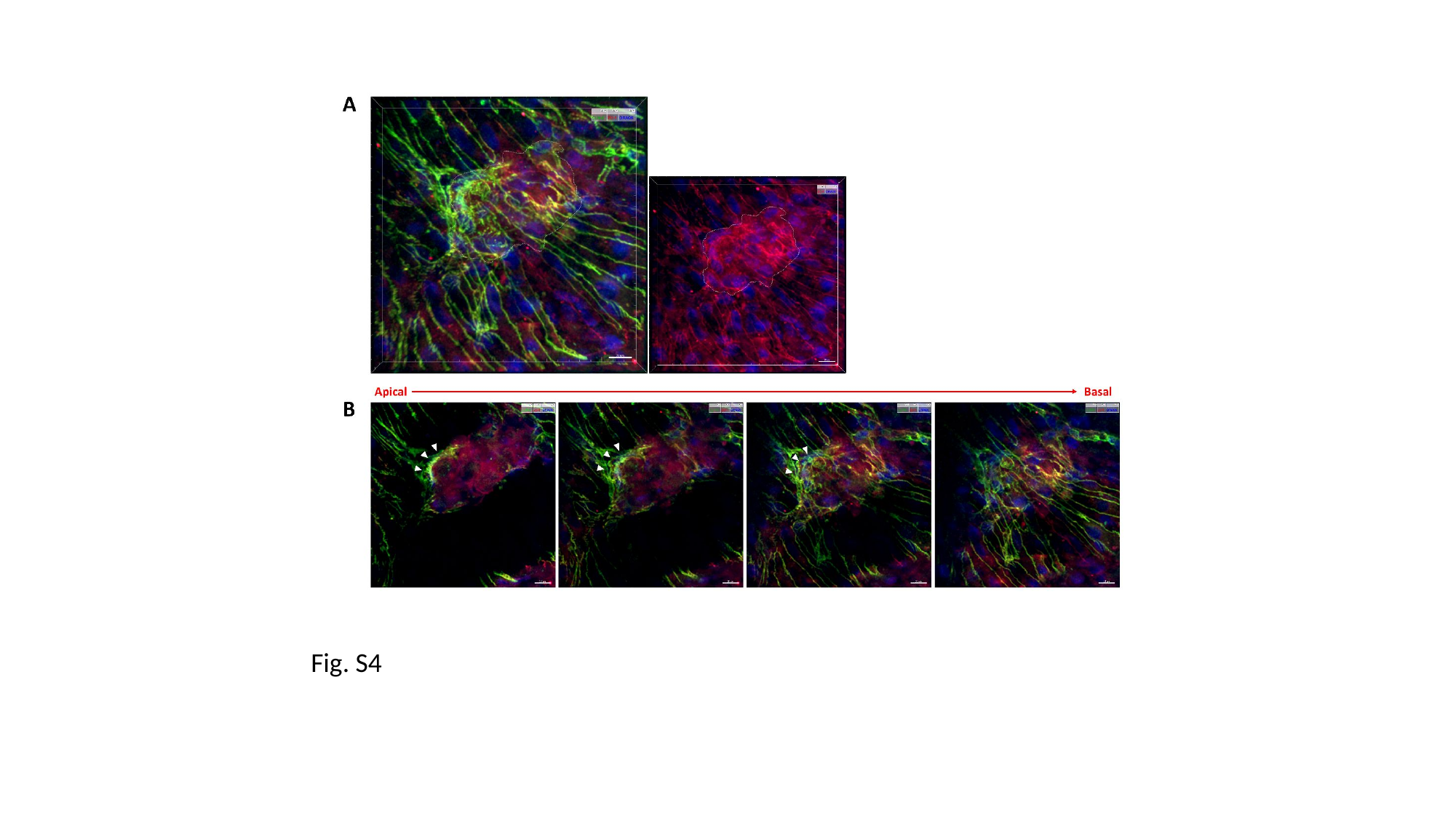

Fig. S4

Supplement: Supplementary file 2 — Additional file 2: Fig. S1. TEER value of BMECs is not passively altered by hES-MSCs. BMECs were cultured on Transwell filters and, following their achieving confluence, hES-MSCs applied, as in Fig. 1 (except no TNF-α was added). After 24 h, TEER was measured. Change in TEER following addition of hES-MSCs is reported as x-fold change of control value. Data are presented as mean ± SE. Each experiment consisted of 3 replicates (derived from a single preparation of BMECs) repeated 3 times (each time from a different BMEC preparation), for a total N = 9 samples per group. No significant difference was detected. Fig. S2. Immunostaining of occludin. BMECs were plated on 24-well Transwell inserts, allowed to achieve confluence, and then (±) exposed to 10 ng/ml TNF-α added to both the bottom and top chamber for 24 h at 37 °C. BMECs were fixed with 4% paraformaldehyde, and then immunostained for the TJ protein occludin. Fig. S3. TJ protein/gene expression in hES-MSCs and BM-MSCs. A. hES-MSCs and BM-MSCs were grown in the 8-well chamber slides coated with 0.1% gelatin. At confluence, hES-MSCs and BM-MSCs were fixed with 4% paraformaldehyde, and then immunostained for TJ proteins CLN-5, ZO-1 and occludin. B. hES-MSCs and BM-MSCs, grown as described in A, were subect to total RNA extraction for relative measurement of CLN-5, ZO-1 and occludin mRNA by qRT-PCR (B). Data are presented as mean ± SE.. Each experiment consisted of 3 replicates (derived from a single preparation of MSCs) repeated 3 times (each time from a different MSC preparation), for a total N = 9 samples per group. Ct values, and not relative expression values are reported, as Ct values for BM-MSC CLN-5 and occludin mRNA were > 35 and, thus, not considered detectable. *p < 0 0.01 compared with hES-MSC group. Fig. S4. Aggregates of hES-MSCs interact with BMEC monolayer. BMECs were plated on 24-well Transwell inserts and allowed to achieve confluence. Thereafter, hES-MSCs were added to top chamber for 24 h, and chem [file 12987_2019_138_MOESM2_ESM.pptx]
